# Whole-Genome Sequence of Pseudomonas frederiksbergensis Strain A6, Isolated from the Rhizosphere of Pepper (Capsicum annuum L.)

**DOI:** 10.1128/mra.00229-23

**Published:** 2023-06-26

**Authors:** Tino Bashizi, Min-Ji Kim, GyuDae Lee, Setu Bazie Tagele, Jae-Ho Shin

**Affiliations:** a Department of Applied Biosciences, Kyungpook National University, Daegu, Republic of Korea; b NGS Core Facility, Kyungpook National University, Daegu, Republic of Korea; DOE Joint Genome Institute

## Abstract

This research presents the whole-genome sequence of Pseudomonas frederiksbergensis strain A6, which was isolated from the rhizosphere soil of pepper (Capsicum annuum L.). The genome of the strain is composed of a single chromosome with 6,711,706 bp, and the GC content is 58.7%.

## ANNOUNCEMENT

Pseudomonas frederiksbergensis is a Gram-negative bacterium. Many Pseudomonas species are beneficial bacteria that can provide a range of advantages to plants, including improvement of nutrient uptake (e.g., nitrogen fixation) and beneficial engagements with other microbes. In addition to supporting host plant nutrition, they aid in the development of plants by enhancing their tolerance to both biotic and abiotic stress factors (1–3).

The genome sequence of P. frederiksbergensis strain A6 was obtained after isolation on 24 January 2021 from the rhizosphere soil of pepper, which was sampled from the greenhouse of Kyungpook National University (Daegu, South Korea) (33.5651°N, 73.0169°E). Briefly, 1 g of soil rhizosphere was serially diluted up to 6-fold and plated on tryptic soy agar (TSA), followed by incubation for 5 days at 30°C. A colony of the strain was isolated and subcultured repeatedly to yield a single pure colony, which was incubated in tryptic soy broth (TSB) for 24 h prior to molecular identification.

The genomic DNA was extracted using the Wizard genomic DNA purification kit (Promega, USA) in accordance with the manufacturer's instructions. The amount of DNA was quantified with a Qubit 2.0 fluorometer (Thermo Fisher Scientific, USA), while its quality was evaluated with a NanoDrop One/OneC microvolume UV-visible spectrophotometer (Thermo Fisher Scientific). Before generation of the sequencing library, genomic DNA was not subjected to any form of selection. The library was created following the guidelines provided by the manufacturer for the use of the ligation sequencing kit SQK-LSK109 (Oxford Nanopore Technologies [ONT]) with the NEBNext companion module (New England Biolabs, USA). Subsequently, the ONT MinION platform was utilized to sequence the library for 72 h with the aid of a FLO-MIN111 flow cell (R10.3; ONT). To generate FASTQ files, base calling was performed with Guppy v4.4.1 software running in high-accuracy mode. For quality trimming, sequences with Phred scores of <7 were eliminated from subsequent analyses. The sequencing produced a total of 98,584 reads, with an *N*_50_ of 19,358 bp. *De novo* assembly was carried out using Flye v2.8.3-b1695 with default parameters except for the genome size option (–nano-raw –genome-size 6m –threads 72) (4–6).

**FIG 1:**
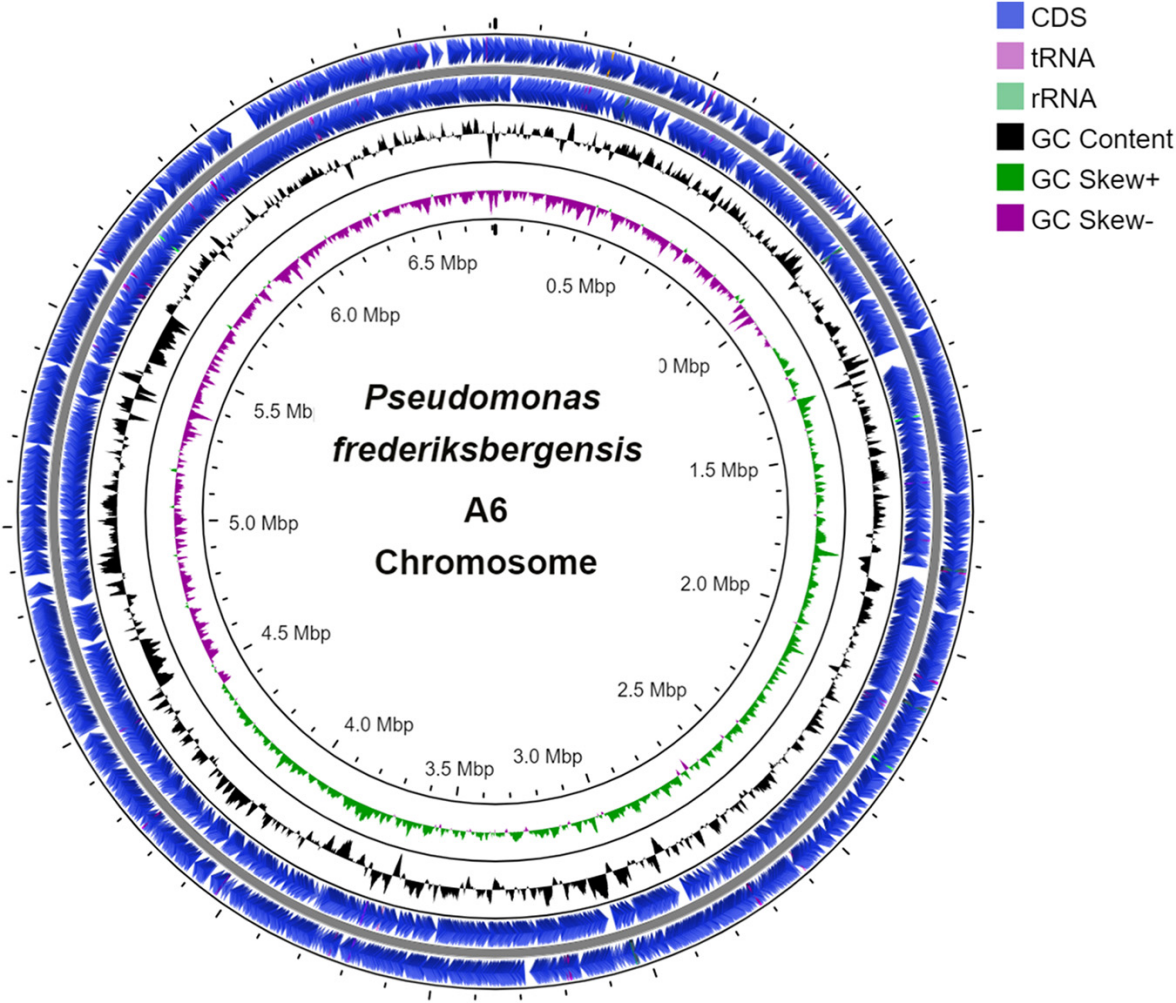
Genome map of the P. frederiksbergensis A6 circular chromosome sequence, generated using the CGView visualization tool.

The genome of P. frederiksbergensis strain A6 was sequenced with a size of 6,711,706 bp consisting of 1 contig, with an *N*_50_ value of 6,711,706 bp and coverage of 138.0×. Verification of the assembly was performed through a Gepard-generated dotplot, while CGView was used to visualize the whole-genome sequence (Fig. 1). Furthermore, the genome was annotated utilizing NCBI PGAP and the RAST server (7). As a result of this process, 5,601 protein-coding genes, 19 ribosomal RNAs, 70 transfer RNAs, 4 noncoding RNAs, and 459 pseudogenes were identified.

### Data availability.

The complete genome sequence data for P. frederiksbergensis A6 have been submitted to the DDBJ/ENA/GenBank database with the accession number CP086236.1. The raw sequencing data are available under the SRA accession number SRR24138994.
